# Chemical Action on Tooth Bone and Fillings

**Published:** 1880-06

**Authors:** G. S. Shattuck


### CHEMICAL ACTION ON TOOTH BONE AND FILLINGS.

BY G. S. SHATTUCK, D. D. S.

A dentist, in the practice of his profession, without some knowledge of chemistry, is like a mariner at sea without a compass or chart of the coasts, or knowledge of the rocks which lay in his course. There are breakers ahead, and he perceives them not until his craft is brought up with a crash, and he is forcibly admonished of his deficiencies. Chemistry teaches us that air, water, earth, in fact every material substance is composed of a few elements, a knowledge of these elements and the various substances or compounds they form when combined. A knowledge of chemical force or affinity, by which these elements are held in combination, is of inestimable value to a man in any profession or calling, but more especially the dentist and physician. Chemistrv teaches us that an element is an indecomposable substance, which always remains the same under all circumstances, although it may enter into many combinations, atom to atom, or otherwise, but still it will retain its identity; as hydrogen and oxygen compose water, $H_2O$. The hydrogen remains hydrogen and the oxygen oxygen, but whenever or wherever they combine in the above proportions, they always form water.

Of the sixty-seven elements known to the world, less than twenty enter into the composition of the human body. There are elements contained in food which enter into the system and form compounds of different natures, upon being digested and assimilated. Each of these elements have their affinities, and when they come in contact with a substance for which they have a greater affinity than the one with which they are combined, they will free themselves and form new combinations. The human system is a chemicai laboratory, where both analysis and synthesis are carried on. One of the important chemical changes which take place is the changing of starch into sugar by the saliva. The production of animal heat is by a mysterious chemical action. The oxygen is received into the system through the lungs, and is thrown out carbonic acid gas; but the process of carbonizing oxygen within the system is not well understood. It is easy to estimate that a given amount of oxygen will produce a definite amount of carbonic acid gas, and will, by its union with carbon and hydrogen, generate a certain number of units of heat; but the mechanism of producing animal heat is too complex and not well enough understood to admit of such positive reasoning. Let some investigating genious discover the exact process of carbonizing oxygen as it takes place in the human system, and utilize it in producing caloric to heat our houses after the fashion our bodies are heated, and a fortune awaits him far greater than that about to be realized by our irrepressible Edison on his electric light.

It is not my purpose to merely define elements, molecules and atoms, or describe chemical affinities and compounds, but to notice some chemical changes which take place in the mouths of our patients, and the effect such changes have upon the teeth. According to chemical analysis, we find the teeth chiefly composed of lime. They have a very dense, hard covering, called enamel, which is the hardest of bone substance. But, notwithstanding its extremely dense and hard nature, we find elements and compounds occurring in the mouth which have a decided chemical action upon it, and which tears it down or dissolves it. Let us examine some of these elements, and the modus operandi of their attacks. First, let us notice the influence of temperature on the teeth. With few exceptions, all bodies expand when their temperature is increased, and contract when it is decreased. Just how this effect is produced we have only a theory, which is, the particles of caloric enter into or among the particles of the body, and partially overcome their cohesion, and cause them to separate farther from each other, and when the particles of heat are withdrawn, the molecules of the body are allowed to approximate each other more closely. The teeth are no exception to this law. When they come in contact with hot substances, the enamel expands, which causes an unpleasant sensation to pass through the tooth; then, upon the immediate application of a colder substance, a contraction takes place, and a pinched sensation is felt, as if the tooth was gripped in a vise. This expansion and contraction cause the enamel to crack or check. These checks in the enamel are more frequently observed in the anterior teeth of the upper jaw, as they are more exposed to thermal changes; but they rarely if ever penetrate deeper than the epidermis of the enamel, and seldom result in caries of the tooth.

But there are other elements which come in contact with the enamel, which act as solvents, and affect its integrity by direct action. The deep fissures in the grinding surfaces of the molars and bicuspeds, and the interstitial spaces between all the teeth, act as recepticles for foreign substances, foods, etc., and in a short time a decomposition or ferment is set up, which changes the combination of the elements contained in the food substance, and form compounds which act readily upon the enamel.

By fermentation, we mean a change or modification which takes place by the presence of a substance called ferment, which acts simply by its presence. Yeast is an active ferment, and is always present in leavened bread. The temperature most favorable to fermentation is from 70° to 100° Fahrenheit. The uniform temperature of the mouth is 98°. Foods of a starchy nature are turned into sugar by the saliva, and greatly assist in promoting fermentation. Animal and vegetable foods, and all foreign substances which remain in the mouth for any length of time, are prone to fermentation and decomposition. The saliva itself is subject to decomposition.

The several different fermentations have received separate names, as vinous or alcoholic fermentation, by which alcohol is produced from starch or sugar; viscous fermention, by which sugar is turned into viscous, or mucilaginous substance, frequently found in ropey saliva, or about the free margin of the gums; the acetic fermentation, by which dilute alcohol is turned into acetic acid. It is not necessary for me to show the process by which acids are formed in the mouth, for indeed we find mouths in such a state of impurity that we would not be surprised to find almost any substance present that could be produced in a chemical laboratory.

In a paper read in this society at a former meeting, I endeavored to show what acids and substances are found in the oral secretions. We will now examine their behavior while in contact with tooth bone. It is not so much the acids which are taken into the mouth in food and drinks that act as solvents, as it is those acids and substances that are formed by chemical combinations, which are constantly taking place in the mouth, that does the mischief. Their constant formation and contact with the teeth, at the temperature of 98°, is the occasion of action on the tooth substance. It is not the amount of damage done in one day or one month, but it is the constant action of the solvent. It would be impossible, in a paper of this kind, to give an accurate description of all the chemical changes which take place in the mouth when the secretions become abnormal; but I will try and give a description of some of the changes that would, and in all probability do occur, where such solvents as has been found in these secretions are present.

The teeth are composed of phosphate, carbonate and fluoride of lime, magnesia phosphate, soda chloride, gelatine and water. The acids usually found in the mouth are hydrochloric, citric, sulphuric, nitric, acetic, oxalic and carbonic. Hydrochloric acid, acting on the teeth, would turn the lime salts into soluble chlorides of lime, except the fluoride of lime, which is only sparingly soluble in hydrochloric and nitric acids ($HCl$ & $HNO_3$) The magnesium (Mg.) and sodium are turned into soluble chlorides. Nitric acid turns them into soluble nitrates. The effect of sulphuric acid ($H_2SO_4$) may be seen in black decay, it decomposes lime salts present, forming insoluble sulphates, soluble Sodium and Magnesic Sulphates, sets free Hydrofluoric Acid, dissolves the gelatinous matter in the teeth and sets free any inorganic matter which comes in its way.

Acetic,<br>Oxalic,<br>Carbonic, } Acids act on the teeth.

Forming respectively Acetates, Oxelates and Carbonates. The action of Carbonic Acid or Carbonic Anhydride upon lime-rock is well known, and may be witnessed in the formation of the Mammoth Cave in Kentucky. The Carbonic Anhydride is formed from decaying vegetation, leaves of the trees &c., it seeps through the fissures in the lime-rock, dissolving it away until it reaches the water veins which course through these rocks and carry off the dissolved portions. Thus it continues to form and dissolve the rock for countless centuries of years, until the enormous subterraneous cavern is formed, which is one of the wonders of the world, all by this chemical action taking place in obedience to the unalterable laws of nature. Having examined the action of acids and compounds on the tooth-bone itself, let us now see what action they would have on the different metals which are used in filling the teeth; this we will consider under the head of Galvanism.

In order to generate a galvanic current, we must have two different metals and a liquid which acts on one of them and not on the other, these must be connected by a conductor. Let us now examine and see if such conditions occur in the mouth. Enough has been said to show that acids form and are found in the mouth in strength sufficient to dissolve the lime salts of the teeth, now let us see what action these would have on the different metals which are used in filling teeth. Of the filling material, we find gold first on the list. This metal, (we are informed by the manufacturers of gold foils,) when prepared for filling teeth it is in an absolutely pure state, therefore it cannot be acted upon by any of the acids except a combination of nitric and hydrochloric, aqua regia, which in all probability would never occur in the mouth in strength to act upon gold. Therefore, we may say that gold as a filling in the teeth is perfectly impervious to all chemical action. Now let us examine some of the baser metals which are used in filling teeth, of these we find tin and amalgams mostly in use. Tin tarnishes very little in pure air, or with moisture, but more in air containing hydrosulphuric acid. It is dissolved by hydrochloric acid, quite slowly when the acid is dilute and cold, quite rapidly when not, and concentrated Stannous Chloride and hydrogen being produced, Dilute Sulphuric Acid acts upon it slowly. Nitric Acid concentrated (according to Prof. Prescott) does not act on tin, but the dilute acid rapidly converts it into meta Stannic Acid ($H^{10}Sn^5O^{15}$) an insoluble compound, (insoluble in acid) very dilute Nitric Acid acts on tin without evolution of gas. Boyl, who wrote in the year 1670, remarks, that aquæfortis eats up or destroys more tin than it disolves, and makes mention of tin becoming gelatinous in aquæfortis. Kuncle, who also studied the action of nitric acid on tin, mentions that tin can only be dissolved when it is added in small quantities to an acid, and that heat must be altogether avoided, because white calx of tin is thrown down when the acid is hot. Roscoe adds, that the explanation of these different statements is to be found in the fact that when this metal is heated with weak nitric acid it forms either stannous or stannic nitrate according to the degree of concentration of the acid; and that the latter salt easily decomposes with seperation of gelatinous stannic acid.

Whilst on the other hand, when tin is acted upon by strong nitric acid it is violently attacted with evolution of heat and formation of an insoluble white powder consisting of meta stannic acid. I have found by actual experiments that dilute or commercial nitric acid acts very rapidly on tinfoil rolled into a rope, with evolution of heat, forming it into a white powder insoluble in water and acid, Except nitro-hydrochloric. On tinfoil condensed into a filling (an old tin filling) the action was much slower with less heat, but the result was the same, while the same in concentrated nitric acid, the action was very slow and the filling of tin soon become coated with a grey white compound and the action of the acid ceased in a short time.

To further test the action of nitric acid on tin, a filling of it was put in a test-tube containing some Saliva which was in neutral condition, enough nitric acid was added to give the whole a slightly acid re-action, this was then placed in a sand bath at 98° Far. and kept there 24 hours and upon examination it was found to be considerably corroded and coated with an insoluble compound. A filling of Prof. Chase's plastic tin was treated in the same way with result of it becoming porous. By the dissolving mercury by the acid; other amalgams were tried with same results, only they became much more porous.

Tin forms two stable oxides: stannous oxide (Sno) and stannic oxide ($Sno_2$), the latter acts as a base in stannic compounds and as anhydride in stannates of metals.

(The fact, that tin readily forms a calx, was observed at an early period.) These oxides are insoluble in water and when they occur in the mouth, would not effect the integrity of the filling. The blackened condition of an old oxidized tin-filling in the mouth is probably due to the sulphur contained in the albumen in the salivia, which is set free as hydrosulphuric acid gas and converts the oxide into a sulphide. Practically we all know, that an oxydized tin-filling protects the tooth bone from decay, as long as it is in contact with the walls of the cavity. There possibly may be a feeble current of electricity between tin and gold in contact with each other, if the saliva is in condition to act very forcibly on the tin. It would practically be of very little importance, as the tin would soon be covered with an insoluble compound; this would cause the wasting of the tin to cease and consequently the current of electricity would cease also.

*Amalgams.* We find numerous metals entering into the composition of amalgams: silver, mercury, tin, cadmium, palladium, copper, gold, &c. If a filling of amalgam is in contact with gold or even in very close proximity with it, I believe that a larger and more prominent current is produced, because mercury, tin, copper, silver, &c., are more easily corroded than gold. In this case, I believe the current may be of sufficient strength and duration to promote decay and otherwise annoy the patient by the galvanic effect on the nerves, if the saliva was in the proper acid condition to act on the filling. With the saliva in normal condition of course there would be no effect. Therefore we often hear practical dentists denying that there is or can possibly be any galvanic action between amalgam and gold filling, because they do not understand, or entirely ignore the connecting medium, viz.: the action of acid saliva; or, as in most cases, they never give the chemical conditions of the oral secretions a passing thought and jump at conclusions based on a normal state of the mouth and its fluids. The chemical action between gold and amalgam in contact would tend to generate potassic and sodic carbonates and sulphides from the saliva. The amalgam would tend to form chloride of the several metals of which it is composed. Either of these classes of bodies would act injuriously on the calcic phosphate of the teeth.

